# Medical Dogs

**Published:** 1899-07

**Authors:** 


"Medical Dogs."—The latest solecism in medical literature I find,—much to my surprise,—in the *Journal of the American Medical Association*. The editor, in speaking of a recent exhibition of dogs belonging to physicians and physicians' families, calls it a "medical dog show." Now, whether he means by that that the dogs were "medical," or that it was a "medical" show of *lay* dogs, I don't know. This is a barbarism of which Dr. Simmons would hardly have been thought capable. The editor of the *National*

*Medical Journal* should set a better example. In what sense do the dogs of a doctor "pertain to the art of healing disease?" (that is the definition of "medical"), or in what sense does a "show" of doctors' dogs pertain to the said art? If a doctor's belongings are "medical," we shall soon hear of "medical horses," and should not be surprised any day to see a poster in front of the popular drug store: "Hitch none but medical horses here." Then, too, the doctor's wife becomes "medical," and his man-servant, his maid-servant, and his ox and his ass and everything that is his; and by a stretch of courtesy the stranger within his gate might become "medical" also.

Now, it must be a great thing for a dog to be a "medical dog." One is inclined to wonder if the other dogs,—the curs of low degree, don't call him "Doc," and ask him, in dog-talk, what's "good for," say, fleas,—for instance. One would speculate also on the probability of the "medical dog's" looking wise, and regaling all hearers with learned accounts of his "interesting cases," as do some of his (biped) confreres; and would hope, at least, that he did it in much better English than some of the aforesaid biped "medicals" do.

If this thing is not stopped the proud title, "Doc," will soon lose all its dignity, and everything, animate and inanimate, belonging to a medical man or his family will become "Docs." The learned editor of the *Journal American Medical Association* does not exactly throw physic to the dogs, but he dubs them with the title "medical;" the next thing they will be getting a diploma and coming to Texas to practice; everything "goes" here.

There is another vulgarism very common in medical writing. It is the almost universal habit of speaking of one's patient as "male" and "female," e. g., "M. A., female, aged 23," or "J. B., male, aged 34." The males and females of the *genus homo,* species, *bi-manna,* are specifically denominated "man" and "woman," and when either is referred to this distinction should be made. In reading some "clinical reports" where "males" and "females" are spoken of, one is disposed to speculate whether or not the "doctor" might not be a veterinarian, and the "patient" a cow or a bull, as the case may be, or even a William or a Nancy goat; or, indeed, if it might not after all, pan out, upon close inquiry, to be a cat, either of the Thomas or the Tabby persuasion.

But,—what's the use? A fellow said to me the other day: "Dan'els, beware of the fate of the reformer," (citing cases, as the lawyers say).

